# Recovery and prognostic values of myocardial strain in acute anterior and non-anterior wall myocardial infarction

**DOI:** 10.1371/journal.pone.0282027

**Published:** 2023-02-17

**Authors:** Jiali Wang, Ying Kong, Jianning Xi, Min Zhang, Yuan Lu, Chunfeng Hu, Kai Xu

**Affiliations:** 1 Department of Radiology, The Affiliated Hospital of Xuzhou Medical University, Xuzhou, China; 2 Department of Cardiac Care Unit, The Affiliated Hospital of Xuzhou Medical University, Xuzhou, China; Cairo University Kasr Alainy Faculty of Medicine, EGYPT

## Abstract

**Background:**

To assess the recovery and prognostic values of myocardial strain using cardiac magnetic resonance (CMR)- feature tracking (FT) in acute anterior and non-anterior wall myocardial infarction.

**Methods:**

103 reperfused patients after STEMI who underwent CMR at about 4 days (baseline) and 4 months (follow-up) were included, including 48 and 55 patients with anterior wall myocardial infarction (AWMI) and non-anterior wall myocardial infarction(NAWMI). CMR-FT analysis was performed using cine images to measure LV global radial, circumferential, and longitudinal peak strains (GRS, GCS, and GLS, respectively). Infarct size (IS) and microvascular obstruction (MVO) were estimated by late-gadolinium enhancement imaging. The primary clinical endpoint was the occurrence of major adverse cardiac events (MACE) after infarction.

**Results:**

Patients with AWMI had higher IS, higher MVO, lower ejection fraction, and more significantly impaired CMR-FT strain values than patients with NAWMI (all p<0.05). Global strain significantly improved at 4 months (all p<0.01), especial in NAWMI. GLS was an independent predictor (odds ratio = 2.08, 95% confidence interval = 1.032–4.227, p = 0.04] even after adjustment for IS and MVO. The optimal cutoff of GLS was -7.9%, with sensitivity and specificity were 73.3% and 75.0%, respectively. In receiver operating characteristic analysis, IS remained the strongest predictor (area under the curve [AUC] = 0.83, p<0.01), followed by MVO (AUC = 0.81, p<0.01) and GLS (AUC = 0.78, p<0.01).

**Conclusion:**

CMR-FT-derived global myocardial strains significantly improved over time, especial in NAWMI. GLS measurement independently predicted the occurrence of medium-term MACE.

## Introduction

ST-elevation myocardial infarction (STEMI) is an acute emergency that requires prompt reperfusion. Despite improvements in modern interventional and pharmacologic therapeutic treatment, some patients have unfavorable prognoses; therefore, valid risk stratification is crucial for STEMI [1,2]. Various clinical, electrocardiographic, serologic, echocardiographic, and cardiac magnetic resonance (CMR) imaging parameters have been evaluated to predict long-term outcomes early in the postreperfusion period. Left ventricular (LV) remodeling after acute STEMI is significant in the progression of cardiac insufficiency to heart failure, and adverse LV remodeling is associated with poor outcomes [3]. Myocardial strain analysis including the global radial, circumferential, and longitudinal strains (GRS, GCS, and GLS, respectively) is performed using speckle-tracking echocardiography; it describes the relative change in lengths of myocardial segments and reflects both systolic and diastolic LV function. More recently, CMR feature-tracking (FT) has been applied to routine CMR cine sequences without additional scanning time, and this has become a promising standard technique for quantifying myocardial strain in STEMI patients [4,5].

Previous studies showed that patients with acute anterior wall myocardial infarction (AWMI) suffered more pronounced adverse LV remodeling and major adverse clinical events (MACE) than those with non-anterior wall myocardial infarction (NAWMI) [2,6,7]. However, some studies also showed no infarct location-dependent differences in mortality [8]. The purpose of this study was to evaluate how myocardial infarction (MI) location affects myocardial dysfunction and to evaluate the value of myocardial strain parameters (GRS, GCS, and GLS) assessed by CMR-FT in predicting medium-term MACE after primary percutaneous coronary intervention (PCI) in patients with acute STEMI.

## Methods

### Study population

This retrospective study was approved by our hospital’s local ethics committee (No.XYFY2021-KL086-01), the study complied with the Declaration of Helsinki, and informed consent was waived by the committee because of the retrospective nature of this study. STEMI was defined on the basis of a history consistent with acute myocardial ischemia (prolonged chest pain lasting <12 h), ST-segments elevation on the ECG and elevated troponin I concentration. Patients with prior MI or revascularization, hemodynamic instability, atrial fibrillation, significant valvular heart disease, severe renal insufficiency, and conventional contraindication to CMR were excluded. All participants underwent emergent primary PCI and stent placement within 24 h of symptoms onset successfully.

First-time acute STEMI who underwent primary PCI and two CMR examinations—one at day 4 (baseline) and the other at 4 months (follow-up)—after MI between September 2019 and March 2021 were included. The clinical endpoints were assessed by telephone interviews at 3, 6, and 12 weeks and every 6 months thereafter. The primary endpoint of the study was the occurrence of MACE, defined as recurrent angina pectoris, acute myocardial infarction, heart failure, and death due to coronary heart disease.

### CMR protocol

CMR scans were performed on a 3.0T MR scanner (Ingenia, Philips Healthcare, Amsterdam, The Netherlands). All images were acquired with electrocardiographic gating and breath holding. The protocol included steady-state free precession (SSFP), T2-weighted sequences, rest perfusion scans (with an intravenous infusion of 0.15 mmol/kg gadolinium-based contrast agents at an injection rate of 3 mL/s, followed by a 30-mL saline flush), and late-gadolinium enhancement (LGE) sequence 10–15 min after the administration of contrast agents. Both SSFP cine and LGE MR images were obtained including two-, three-, and four-chamber view images and a set of short-axis images covering the entire left ventricle from the atrioventricular ring to the apex. The SSFP sequence was obtained with 30 phases in the cardiac cycle; the parameters were as follows: field of view, 350*350 mm; repetition time/echo time (TR/TE), 2.6/1.3 ms; flip angle, 45°; and slice thickness, 8 mm. The LGE-CMR parameters were as follows: TR/TE, 3.0/ 6.1 ms; flip angle, 25°; and slice thickness, 8 mm.

### CMR data analysis

Anonymized CMR images were analyzed offline with a commercially available workstation (Circle Cardiovascular Imaging, cvi42®, v5.12.4, Calgary, Alberta, Canada) by two cardiac radiologists with 8- and 10- years’ experience respectively blinded to patient history and clinical outcome. The endocardial and epicardial borders were delineated manually in end diastolic images, wherein the software automatically tracks through the cardiac cycle.

**Fig 1:**
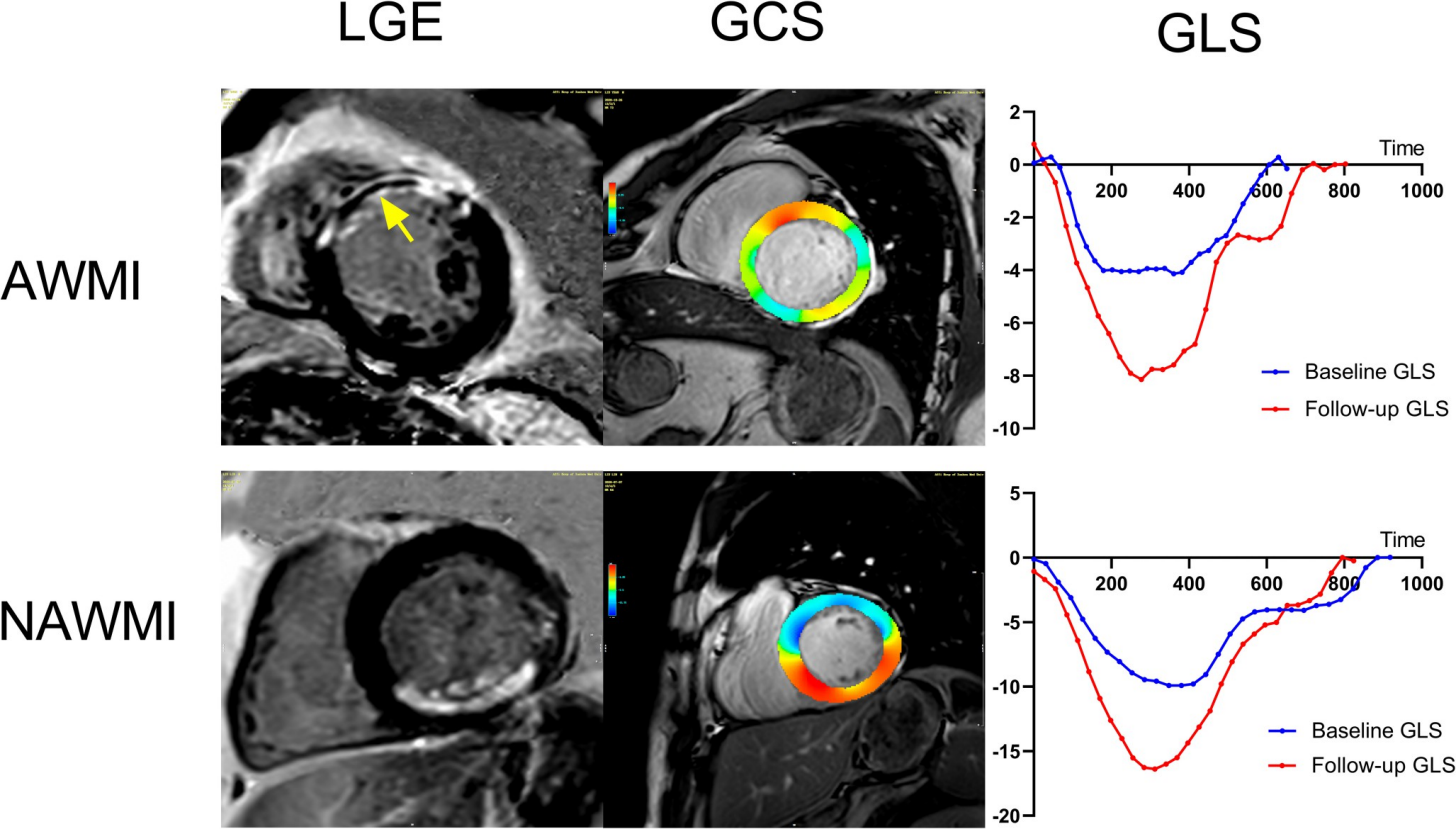
Cardiac magnetic resonance (CMR)-late gadolinium enhancement (LGE) and feature tracking (FT). First line: LGE-CMR reveals anterior wall myocardial infarction (AWMI) with microvascular obstruction (yellow arrow). Second line: LGE-CMR reveals inferior wall myocardial infarction. Pseudo-colored images showed globe circumferential strain (GCS) of the infarcted myocardium was significantly decreased (red part). Globe longitudinal strain (GLS) curves derived from CMR-FT analysis displayed the values of baseline and follow-up global peak systolic longitudinal strains, respectively.

CMR-FT strain analysis was performed on four cine views, namely the two-, three-, four-chamber, and short-axis cine CMR images, and the peak value of radial strain, circumferential strain, and longitudinal strain was set as the GRS, GCS, and GLS respectively for statistical analysis. Left ventricular end-diastolic volume (LVEDV), left ventricular end-systolic volume (LVESV), and left ventricular ejection fraction (LVEF) were assessed from the short-axis cine images. MI represents the region of positive LGE, was automatically identified as the myocardium with signal intensity > 5SD mean signal intensity of remote myocardium. Manual delineation was used to quantify areas of hypointensity within the positive LGE, which represented microvascular obstruction (MVO) (Fig 1). The infarct size (IS) and MVO are expressed as % of LV myocardium for statistical analysis. Anterior wall myocardial infarction was defined as the left main coronary artery or anterior descending branch lesion.

### Statistical analysis

Continuous variables are expressed as mean and SD or as median and interquartile range (IQR), while categorical variables are expressed as counts and percentages. For continuous variables, differences between the two groups were compared using Student’s t-tests or Mann–Whitney U tests, and categorical variables were compared by using the Fisher’s exact tests. Univariate and stepwise multivariate logistic regression analysis were used to identify predictors of MACE. Multivariate regression was performed using only variables that were p<0.1 in univariate regression analyses. Receiver operating characteristic (ROC) analysis was used to evaluate the diagnostic accuracy of baseline strain parameters, IS, and MVO to predict the occurrence of outcome events at the clinical follow-up, the optimal cut-off values were identified by the Youden Index. All statistical analyses were performed with SPSS (version 23.0, Statistical Package for the Social Sciences, IBM Corporation, Armonk, NY, USA), and p<0.05 was considered statistically significant. The collinearity of the parameters is verified by variance inflation factor (VIF), and VIF < 5 indicates that there is no collinearity.

## Results

### Clinical characteristics

A total of 103 patients with both baseline and follow-up CMR scans were enrolled in this study, including 48 and 55 patients with AWMI and NAWMI, respectively. The median age of the overall study group was 55 years (IQR: 47–65 years), and 87 (84.5%) patients were male. The baseline characteristics of the patients with AWMI and NAWMI are summarized in Table 1. There were no significant intergroup differences with respect to age, sex, body surface area, hypertension, smoking status, hyperlipidemia, diabetes, or N-terminal pro–brain natriuretic peptide (NT-proBNP) level of 24–48 hours after onset of acute MI. However, 24–48 hours peak creatine kinase-myocardial band (CK-MB) and high-sensitivity cardiac troponin-T (hs-cTnT) levels were significantly higher in patients with AWMI than NAWMI (p = 0.01 and p = 0.04, respectively). The heart rate was lower in NAWMI than AWMI patients (p = 0.01).

**Table 1:** Baseline characteristics and cardiac magnetic resonance parameters of study population.

Characteristic	All patients (n = 103)	NAWMI (n = 48)	AWMI (n = 55)	p value
Male, n (%)	87 (84.5%)	41 (85.4%)	46 (83.6%)	0.80
Age, years	55±12	57±11	53±13	0.14
Body surface area, /㎡	1.9±0.2	2.2±1.9	1.9±0.2	0.35
Hypertension, n (%)	63 (61.2%)	29 (60.4%)	34 (61.8%)	0.88
Cigarette smoking, n (%)	36 (35.0%)	19 (39.6%)	17 (30.9%)	0.36
Hyperlipidemia, n (%)	52 (50.5%)	24 (50.0%)	28 (50.9%)	0.93
Diabetes mellitus, n (%)	47 (45.6%)	23 (47.9%)	24 (43.6%)	0.66
Clinical follow-up, days	489±143	497±139	481±147	0.44
Heart rate, beats/min	71±9	68±6	74±10	0.01
Serologic tests				
24–48 h hs-cTnT concentration, ng/L	4425±6030	3054±1976	5622±7883	<0.01
24–48 h CK-MB concentration, U/L	236±228	171±156	293±264	0.04
24–48 h NT-proBNP, pg/mL	1049±1299	883±1144	1193±1414	0.40
CMR parameters				
Day of baseline CMR, day	5±2	5±1	5±1	0.37
Baseline LVEF, %	49.6±11.0	52.6±10.6	47.1±10.7	0.07
Baseline LVEDV, mL	151.3±34.4	146.2±29.3	155.6±38.1	0.16
Baseline LVESV, mL	77.7±29.4	70.7±25.9	83.9±31.1	0.03
Baseline IS, % of LV myocardial mass	28.0±11.2	25.2±9.8	30.5±11.8	0.03
Baseline MVO, % of LV myocardial mass	0.8±11.2	0.5±0.9	0.9±1.2	0.01

Values are presented as mean ± standard deviation or n (%). *AWMI* anterior wall myocardial infarction, *CK-MB* creatine kinase-myocardial band, *CMR* cardiovascular magnetic resonance, *GCS* global circumferential strain, *GLS* global longitudinal strain, *GRS* global radial strain, *hs-cTnT* high-sensitivity cardiac troponin T, *IS* infarct size, *LVEDV* left ventricular end-diastolic volume, *LVEF* left ventricular ejection fraction, *LVESV* left ventricular end-systolic volume, *MVO* microvascular obstruction, *NAWMI* non-anterior wall myocardial infarction, *NT-proBNP* N-terminal pro–brain natriuretic peptide. Normal cutoffs: CK-MB<5 ng/mL, hs-cTnT<14.0 ng/L, NT-proBNP ≤450pg/mL.

### CMR findings

In the baseline CMR examination, a significant difference was observed in the LVEDV, LVEF, myocardial IS, MVO and all three LV strain parameters (all p<0.01) between the AWMI and NAWMI groups (Table 1).

Follow-up CMR was completed at a median of 114 days (IQR: 100–123 days) after infarction. The GRS, GCS, and GLS all significantly improved from baseline to 4 months follow-up (ΔGCS: 2.8±5.8%, p<0.01; ΔGCS: -1.2±2.7%, p<0.01; ΔGLS: -1.1±2.0%, p<0.01), and there was a certain effect of infarction location (AWMI vs. NAWMI) on GCS and GLS recovery (ΔGCS: -0.8±3.0% vs. -1.8±2.3%, p<0.01; ΔGLS: -0.8±2.3% vs. -1.5±1.5%, p = 0.01) (Table 2).

**Table 2:** Recovery of global strain in AWMI versus NAWAMI groups.

	Total	p value	NAWMI	AWMI	p value
GRS (%)					
Baseline	21.0±6.0		22.9±6.3	19.5±5.4	0.011
Follow-up	23.8±7.5		25.4±7.4	22.4±7.5	0.051
Change	2.8±5.8	<0.01	2.6±5.8	3.0±5.9	0.83
GCS (%)					
Baseline	-14.5±2.9		-15.9±2.4	-13.3±2.8	<0.01
Follow-up	-15.8±3.4		-17.7±2.4	-14.1±3.3	<0.01
Change	-1.2±2.7	<0.01	-1.8±2.3	-0.8±3.0	<0.01
GLS (%)					
Baseline	-8.5±2.3		-9.2±2.0	-7.8±2.4	0.01
Follow-up	-9.6±2.5		-10.7±1.9	-8.6±2.5	<0.01
Change	-1.1±2.0	<0.01	-1.5±1.5	-0.8±2.3	0.01

AWMI: Anterior wall myocardial infarction, GRS: Global radial strain, GCS: Global circumferential strain, GLS: Global longitudinal strain, NAWMI: Non-anterior wall myocardial infarction.

### Utility of CMR findings for prognostic prediction

At a median clinical follow-up of 18 months (IQR: 12–20 months), 15 patients (8 AWMI and 7 NAWMI) presented with MACE: recurrent MI (n = 9), repeated revascularization procedures by PCI (n = 5), and coronary artery bypass surgery (n = 1). There was no difference in the incidence rates of MACE between the AWMI and NAWMI groups. All characteristics in Table 1 were analyzed using univariate logistic regression analyses, and baseline LVEDV, LVESV, LVEF, IS, MVO, GLS, GRS, and GCS showed significant predictive associations with the development of MACE. However, in the multivariable analysis, only IS (odds ratio [OR]: 1.23 [1.05–1.45], p = 0.012); MVO (OR: 3.68 [1.36–9.98], p = 0.011); and GLS (OR: 2.09 [1.03–4.23], p = 0.04) were independent predictors of MACE (Table 3). VIF of IS, MVO, GLS were 1.05, 1.06, 1.01 respectively, indicating that the model we constructed did not be affected by collinearity.

**Table 3:** Logistic regression analysis for the prediction of adverse cardiac events.

	Univariate			Multivariate		
	OR	95% CI	p value	OR	95% CI	p value
CK-MB,U/L	0.99	0.999–1.001	0.10	0.99	0.97–1.02	0.02
Baseline LVEDV, mL	1.02	1.00–1.04	0.01	-	-	-
Baseline LVESV, mL	1.04	1.02–1.07	<0.01	-	-	-
Baseline LVEF, %	0.84	0.77–0.92	<0.01	-	-	-
Baseline IS, %	1.16	1.07–1.25	<0.01	1.23	1.05–1.45	0.012
Baseline MVO, %	2.49	1.53–4.08	<0.01	3.67	1.36–9.98	0.011
Baseline GRS, %	0.83	0.73–0.94	0.002	-	–	-
Baseline GCS, %	1.39	1.12–1.72	0.003	-	–	-
Baseline GLS, %	1.67	1.24–2.23	0.001	2.09	1.03–4.23	0.042

CI confidence interval, *CK-MB* creatine kinase-myocardial band, GCS global circumferential strain, GLS global longitudinal strain, GRS global radial strain, IS infarct size, LVEDV left ventricular end-diastolic volume, LVEF left ventricular ejection fraction, LVESV left ventricular end-systolic volume, MVO microvascular obstruction, OR odds ratio.

On the basis of area under the curve (AUC), baseline IS was the most accurate diagnostic parameter for MACE (AUC = 0.832, 95% confidence interval(95% CI) = 0.731–0.932, p = 0.001), then AUC (MVO) = 0.812, 95% CI = 0.685–0.939, p<0.001, AUC (GLS) = 0.779, 95% CI = 0.644–0.914, p<0.001. The optimal cutoff values of GLS for MACE predictor was -7.9%, with sensitivity of 73.3% and specificity of 75.0%.

## Discussion

The present study showed that AWMI patients suffer larger IS and MVO and worse EF and LV global strain than NAWMI patients. Global strains improved over time, especial in NAWMI. All three global strain measures (GLS, GRS, and GCS) were associated with increased MACE rates; however, only GLS was an independent predictor of medium-term adverse clinical outcomes after MI. Further, IS remained the strongest predictor for medium-term MACE.

According to the guidelines for the management of acute STEMI [2,9], anterior infarct location is one of the independent predictors of early death from STEMI. Hand et al. found the early (day 1–28 after MI) and late mortality of AWMI were greater in the anterior MI than inferior MI group [8]. The present study demonstrated that AWMI patients have larger IS and MVO and worse EF, which are consistent with existing literature [6,7,10], However, it remains controversial whether the location of MI is associated with poor prognosis after infarction, or whether the size of MI is more important than the anterior location of the infarction. In our opinion, larger IS always indicates greater myocardial necrosis and more serious myocardial dysfunction, and our univariate analysis showed that anterior MI location was not associated with MACE at medium-term clinic follow-up. Therefore, it is necessary to focus on IS and MVO in acute MI patients regardless of the MI location.

What’s more, both baseline and follow-up GRS, GCS, and GLS of AWMI were lower than NAWMI, and the recovery of GCS and GLS from baseline to 4 months later showed statistically significant differences between the AWMI and NAWMI groups. NAWMI patients showed better recovery than AWMI patients after MI. GCS and GLS are generally negative values by definition, and patients with less circumferential and longitudinal malformation always have a higher risk of adverse remodeling. Myocardial strain can be used to assess both global systolic function and systolic and diastolic function. Strain has substantial potential to be of diagnostic and prognostic value, and most of the clinical evidence for strain measurement is obtained by echocardiography [11]. However, inadequate image quality and considerable observer variability are well-known limitations of echocardiographic techniques and affect myocardial strain measurement reliability [4]. CMR-FT strain parameters can be acquired together with all other standard CMR parameters during a single noninvasive examination without prolonging scan time. Previous studies have shown that CMR-FT strain values demonstrated excellent intra- and interobserver reproducibilities [12–14].

To the best of our knowledge, there is no consensus regarding the association of CMR-FT parameters (GLS, GRS, and GCS) with the highest risk of severe clinical events in the postinfarction period [15–20]. In the current univariate logistic regression, all three global strain measures were associated with the occurrence of MACE, while in the multivariate logistic regression, only GLS was identified as an independent predictor of MACE after adjusting for other established prognostic risk factors including IS and MVO. GLS has strong prognostic value for the occurrence of MACE [15,17]; however, its underlying cause has not been fully elucidated. Myocardial fibers are composed of three layers: the subendocardium are longitudinal fibers, the middle layer are oblique fibers, and the subepicardium are circumferential fibers. When MI occurs, subendocardial myofibers, that are longitudinal fibers, are more vulnerable to early myocardial damage, followed by oblique fibers and circumferential fibers. Therefore, diminished GLS is always an early marker of LV dysfunction. However, Buss et al. studied 74 STEMI patients and demonstrated that the GCS-rather than GLS-provided objective assessment of IS and could predict functional recovery [16]. The reasons for these different findings may be the heterogeneous patient populations with different infarct locations and extents, or the differential effects of longitudinal and circumferential strains on subendocardial infarction and transmural infarction. Besides, different centers with different CMR vendors could have also influenced the data, different field strengths of the machine may influence the results. Mangion et al [21] studied 89 healthy volunteers without cardiovascular disease who underwent CMR at 1.5T and 3.0T within 2 days and found the magnitudes of GLS was greater at 1.5T than at 3.0T. This may be one of the reasons why the cut-off value for GLS to predict MACE in our study was -7.9%, lower than in other studies.

IS and MVO measured by LGE are known to have added value as predictors of adverse outcomes after acute MI [22–24]. Moreover, our results showed that IS surpassed MVO and GLS remained the strongest predictor for MACE. Another prospective observational study on 451 revascularized STEMI patients showed that GLS strongly and independently predicted the occurrence of medium-term MACE, and its prognostic value was superior to IS and MVO [13]. Hence, a comprehensive CMR approach that includes CMR markers of infarct severity such as IS, MVO, and GLS could significantly improve STEMI patient risk assessment. Furthermore, as a contrast agent-free technique, CMR-FT may have the potential as an independent predictor of adverse cardiac events in patients with contraindications to contrast agents, especially those with severe renal impairment.

### Limitations

Our study has some limitations. First, we only included patients with hemodynamically stable and first-time STEMI; no critically ill patients were included. Thus, our results may not be generalizable to hemodynamically impaired, recurrent MI or non-STEMI patients. Second, this was a single-center study and the number of events was low, so further validation is required. Third, the majority of evidence and clinical experience regarding myocardial strain measures have been obtained by echocardiography in the past, however, in this study, due to the lack of echocardiography data in some patients, echocardiography indicators were not included in the study, this is also the disadvantage of retrospective study. Last, quantitative native T1 mapping, postcontrast T1 mapping, and extracellular volume quantification were not used in our study, which have been reported for improving post-STEMI risk stratification in the last few years.

## Conclusion

AWMI patients suffer worse LV global strain recovery than NAWMI, Acute IS, MVO and GLS measured by CMR-FT can independently predict medium-term MACE.

## Supporting information

S1 Data (XLSX)
